# Long non-coding RNAs in ovarian granulosa cells

**DOI:** 10.1186/s13048-020-00663-2

**Published:** 2020-06-05

**Authors:** Jiajie Tu, Yu Chen, Zhe Li, Huan Yang, He Chen, Zhiying Yu

**Affiliations:** 1 Department of Gynecology, The First Affiliated Hospital of Shenzhen University, Health Science Center, Shenzhen Second People’s Hospital, 3002 Sungang West Road, Futian District, Shenzhen, 518000 Guangdong province China; 2 Key Laboratory of Anti-Inflammatory and Immune Medicine, Ministry of Education, Anhui Collaborative Innovation Center of Anti-Inflammatory and Immune Medicine, Institute of Clinical Pharmacology, Anhui Medical University, 81 Meishan Road, Hefei, 230032 Anhui province China

**Keywords:** GCs, lncRNA, Folliculogenesis, PCOS, POI

## Abstract

Granulosa cells (GCs) are somatic cells surrounding oocytes within follicles and are essential for folliculogenesis. Pathological changes in GCs are found in several ovarian disorders. Recent reports have indicated that long non-coding RNAs (lncRNAs), which modulate gene expression via multiple mechanisms, are key regulators of the normal development of GCs, follicles, and ovaries. In addition, accumulating evidence has suggested that lncRNAs can be utilized as biomarkers for the diagnosis and prognosis of GC-related diseases, such as polycystic ovary syndrome (PCOS) and premature ovarian insufficiency (POI). Therefore, lncRNAs not only play a role in GCs that are involved in normal folliculogenesis, but they may also be considered as potential candidate biomarkers and therapeutic targets in GCs under pathological conditions. In the future, a detailed investigation of the in vivo delivery or targeting of lncRNAs and large-cohort-validation of the clinical applicability of lncRNAs is required.

## Introduction

Granulosa cells (GCs) are somatic cells of the sex cord [1], which are associated with the development of oocytes in mammalian ovaries [2, 3]. In addition, GCs are also implicated in various ovary-related diseases, including polycystic ovary syndrome (PCOS) [4], premature ovarian insufficiency (POI, also referred to as premature ovarian failure [POF]) [5], ovarian hyperstimulation syndrome (OHSS) [6], and GCs tumor (GCT) [7]. Previous studies have shown that genetic factors are involved in the development of these diseases [8–11]. Recent evidence has shown that several non-coding RNAs also affect these female reproductive system dysfunctions.

Development of sequencing technology has led to in-depth genome and transcriptome analysis, which showed that over 85% of the human genome is transcribed [12]. However, the amount of protein products from RNA transcripts is very low compared to the overall number of transcripts, indicating that most RNA transcripts are non-coding. Such a large number of transcripts of non-coding RNAs (ncRNAs) suggests that ncRNAs play a more important and diverse role in biological processes than initially expected [13, 14].

NcRNAs can be roughly divided into two groups: a group of short RNAs with a length less than 200 nucleotides long, such as microRNAs (miRNAs), small interfering RNA (siRNA), and piwi RNA (piRNA). The other category is long ncRNAs (lncRNAs) with a length longer than 200 nucleotides. Due to the lack of protein-coding capability, non-coding transcripts have been known as “junk DNA” or “transcriptional noise” for the past few decades [15]. However, many recent lncRNA-based studies have proved that lncRNAs have several functions and act via multiple regulatory mechanisms, including decoy, enhancer RNA, scaffold, guider, microRNA sponging and short peptides [16, 17]. Long non-coding RNAs are classified based on their specific characteristics and their position relative to the host or adjacent protein-coding gene. Location-based classification of lncRNAs assigns them into categories such as antisense RNAs, long intergenic non-coding RNAs (lincRNAs), sense overlapping transcripts, sense intronic transcripts, and processed transcripts [18]. Characteristic-based classification, on the other hand, assigns them to categories such as lncRNA-activating (lncRNA-a) genes, pseudogenes, telomere-associated non-coding RNAs (TERRAs), transcribed ultraconserved regions (T-UCRs), enhancer RNAs (eRNAs), and circular RNAs [19–21]. Compared with the multitude of studies performed on miRNAs and protein-coding genes, we are still at a relatively early stage of investigating, naming, classifying, and identifying lncRNAs.

Moreover, emerging studies have demonstrated that some lncRNAs affect the function of ovarian GCs and are thereby involved in both physiological conditions and pathological processes, such as human oocyte maturation, fertilization, embryo development, tumorigenesis, [22] and ovarian failure [23, 24]. These studies have suggested that GC-specific lncRNAs could be considered as candidate diagnostic or prognostic markers, as well as treatment targets for various ovarian diseases [25–27]. In this review, we summarize the roles of lncRNAs in healthy and dysfunctional GCs and discuss the potential utilization of lncRNAs as diagnostic markers or treatment targets in clinical conditions.

## The role of lncRNAs in GCs under physiological condition

### High-throughput studies of lncRNAs in normal GCs

Long non-coding RNAs are extensively transcribed and play a role in a variety of biological functions in the human genome. Communication between GCs and oocytes is essential for oogenesis [28]. Studying lncRNAs in GCs is important for a better understanding of the mechanisms of follicular maturation, fertilization, and embryo selection.

Yerushalmi et al. reported that compact GCs (CGCs) from germinal vesicle cumulus oocyte complexes (COCs) were isolated from patients undergoing in vitro maturation (IVM). Expanded GCs (EGCs) from metaphase 2 COC were isolated from patients undergoing in vitro fertilization (IVF)/intracytoplasmic sperm injection (ICSI). Global transcriptome profiles of CGCs and EGCs were compared, and 89 lncRNAs with significantly altered expressions were identified. Among them, 12 lncRNAs were encoded within introns of genes that were associated with GC development, suggesting a potential involvement of lncRNAs in cumulus expansion and oocyte maturation. Yerushalmi et al. also generated a library of genes regulated during cumulus expansion and oocyte maturation processes using RNA sequencing. Analysis of these genes led to the identification of novel lncRNAs that are potentially involved in COC maturation and cumulus expansion, which might boost our understanding of the process of oocyte maturation and ultimately improve the IVM efficiency [29].

Using microarray analysis, another study compared the expression profiles of lncRNAs in GCs from three pairs of mature oocytes that gave rise to high-quality embryos and poor-quality embryos. A total of 20,563 lncRNAs were identified in GCs. Among them, one hundred and twenty-four lncRNAs were upregulated and five hundred and nine lncRNAs were downregulated in GCs from poor-quality embryos when compared to GCs from high-quality embryos. In addition, Gene ontology (GO) was used to analyze these significantly differential expressed lncRNAs. It is believed that GC-lncRNAs may exert their functions via interactions with coding transcripts and proteins in the development of oocyte and early embryos. Therefore, lncRNAs in GCs may contribute to the process of oogenesis [30].

Another interesting study by Macaulay et al. [31] showed that GCs contribute to the maternal reserves by actively transferring a series of intermediate factors, including lncRNAs, to oocytes. This unexpected exogenous trafficking to the maternal storage offers a new perspective on the determinants of female fertility. However, the exact molecular mechanism of this communication between GCs and oocyte is yet to be revealed. The specific role lncRNAs, in this transferring, warrants further investigation.

Published sequencing data [32, 33] were used to identify the abundant lncRNAs in metaphase II (MII) oocytes and surrounding GCs. The function of the identified lncRNAs was predicted by the expression network of lncRNA-mRNA. Bioinformatic analysis showed that several lncRNAs (NEAT1, MALAT1, ANXA2P2, MEG3, IL6STP1 and VIM-AS1) in GCs are involved in apoptosis and extracellular matrix-related functions, indicating that lncRNAs in GCs were essential for oocytes growth. This study of lncRNAs in human MII oocytes and GCs provided potential biomarkers for the identification of embryos with high development potential [34]. The above-mentioned studies indicated that lncRNAs expression is significantly altered in GCs [29, 30, 34]. These lncRNAs may play an essential role in female reproduction by directly cross-talking with oocytes [31].

### Individual studies of lncRNAs in normal GCs

#### LncRNA-LET

The endogenous expression of LncRNA-LET in KGN cells is quite low. Overexpression of this lncRNA in KGN cells repressed proliferation and migration and induced apoptosis. LncRNA-LET also repressed KGN cell epithelial-mesenchymal transition (EMT) by inducing E-cadherin and reducing N-cadherin and vimentin expression. An essential EMT-associated factor, tissue inhibitor of metalloproteinases 2 (TIMP2), was directly induced by lncRNA-LET. In addition, TIMP2 overexpression generally mimicked the lncRNA-LET function in KGN cells, validating the idea of TIMP2 being downstream target of lncRNA-LET. Moreover, lncRNA-LET and TIMP2 activated the Wnt/β-catenin and Notch pathways in KGN cells. Overall, lncRNA-LET repressed proliferation, migration and EMT, and promoted apoptosis of KGN cells by activating TIMP2 and inducing the Wnt/β-catenin/Notch pathways [35].

#### LncRNA-Amhr2

Anti-Müllerian hormone (AMH) is mainly produced and secreted from GCs and is essential for repressing Mullerian duct development [36]. The long non-coding RNA, lncRNA-Amhr2, which is transcribed from the upstream region of the AMH receptor type 2 (Amhr2), was identified in GCs. Amhr2 expression was inhibited in lncRNA-Amhr2 knockdown mouse primary GCs (the isolated primary mouse GCs are mixed cumulus GCs and mural GCs). In addition, reporter assay results showed that lncRNA-Amhr2 activation induced Amhr2 promoter activity. This direct transcriptional regulation was further validated in a mouse GCs line (OV3121 cells). This study suggested that lncRNA-Amhr2 activated the transcription of its neighboring gene Amhr2 in GCs, demonstrating a novel mechanism of Amhr2 regulation in the female reproductive system [37].

#### LncPrep96kb

Prolyl oligopeptidase (POP) is a serine endopeptidase that is involved in progesterone secretion [38]. To further investigate the regulation of Prolyl oligopeptidase (POP) in GCs, the genomic loci of POP and six neighboring lncRNAs were studied. Since there are two different transcriptional start sites (TSS), the one adjacent to lncRNA-lncPrepþ96kb was transcribed as two isoforms. This lncRNA induced POP in primary ovarian GCs (the isolated primary mouse GCs are mixed cumulus GCs and mural GCs), and both lncPrepþ96kb and POP were up-regulated in the hormone-treated ovaries [39].

#### LncRNA-AK124742

LncRNA-AK124742 was identified as an antisense of proteasome 26S subunit, non-ATPase, 6 (PSMD6). A positive association was found between AK124742 and PSMD6 in cumulus GCs. Up-regulations of AK124742 and PSMD6 were observed in high-quality embryos (HCCs) compared to poor-quality embryos (PCCs). The relative expressions of AK124742 and PSMD6 were up-regulated in the pregnancy group compared to those in the non-pregnancy group. AK124742-PSMD6 is a potential lncRNA-mRNA biomarker in cumulus GCs involved in embryo selection [24].

#### LncRNA-HAS2-AS1

Cumulus expansion is a luteinizing hormone (LH)-mediated ovulatory process. Hyaluronan synthase 2 (HAS2) affects the synthesis of hyaluronic acid (HA), a major component of cumulus expansion. LncRNA HAS2 antisense RNA 1 (HAS2-AS1) expression was low in immature GCs and up-regulated in mature GCs. HAS2-AS1 inhibition caused the suppression of HAS2 and repressed the migration of cumulus GCs, suggesting that ﻿HAS2-AS1 is an LH-targeted gene that modulates cumulus expansion and migration by increasing HAS2 expression [40].

**Fig. 1:**
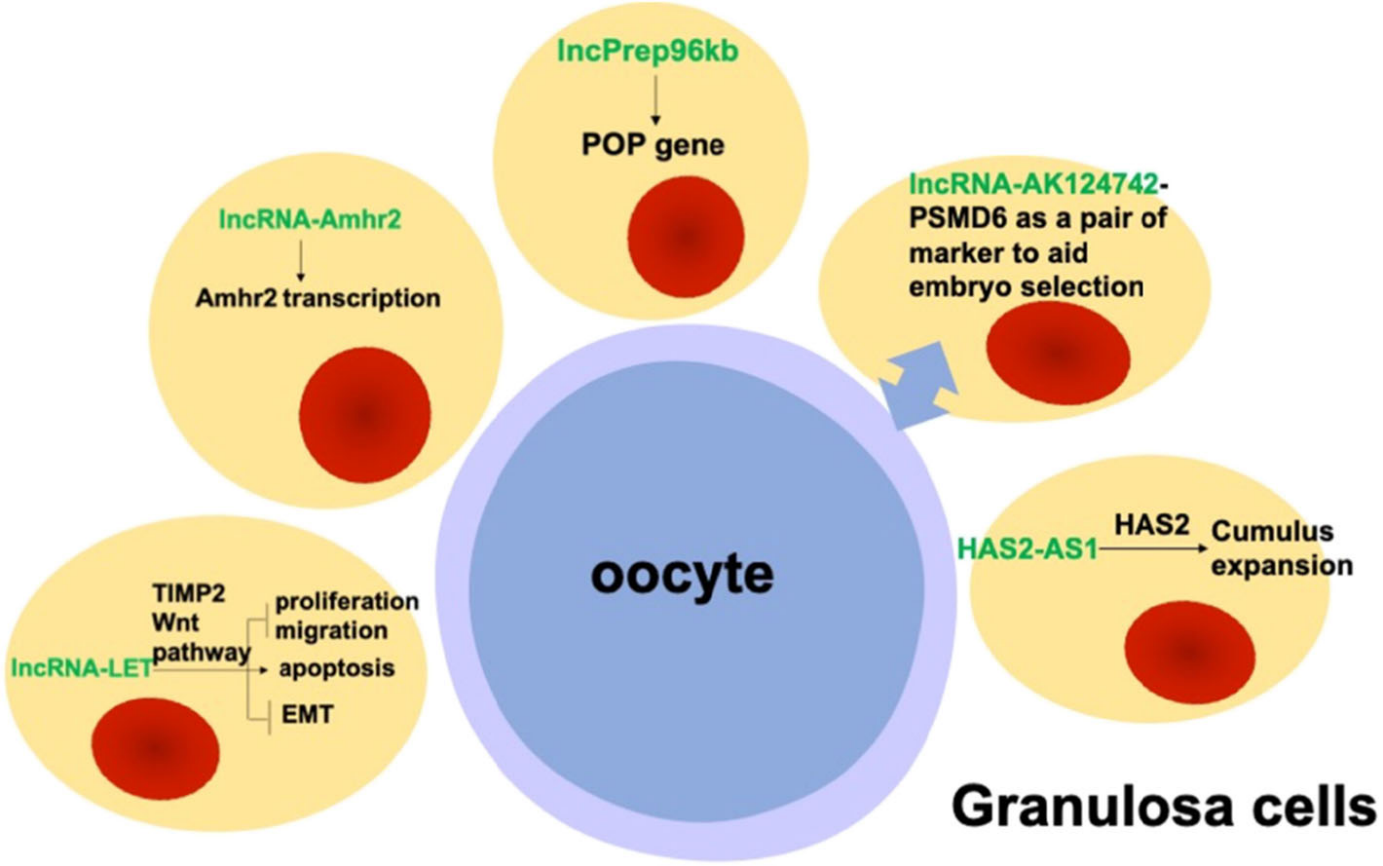
The role of individual lncRNAs in normal granulosa cells (GCs). lncRNA-LET repressed proliferation, migration, epithelial–mesenchymal transition (EMT) and promoted apoptosis in a human GCs line KGN by activating TIMP2 and inducing the Wnt/β-catenin/Notch pathways; lncRNA-Amhr2 activated the transcription of its neighboring gene Amhr2 in primary mural and cumulus GCs and in a mouse GC line OV3121 cells; lncRNA-lncPrepþ96kb induced POP in primary ovarian mural and cumulus GCs; AK124742-PSMD6 is a potential lncRNA-mRNA biomarker in cumulus GCs for embryo selection; HAS2-AS1 caused activation of HAS2 and induced cumulus GCs migration

To summarize, these studies in the above section focused on the roles of five individual lncRNAs in normal GCs, and their findings suggested that lncRNAs could affect the GCs and ovaries via different mechanisms. Although the role of lncRNAs in GCs have not been well-established under physiological conditions, the aforementioned reports show that they play an essential role in GCs. Specifically, lncRNAs (including Amhr2, lncPrep96kb and HAS2-AS1) regulate host or adjacent gene function in GCs. In addition, lncRNAs (such as AK124742) could be potential biomarkers of embryo selection. Individual lncRNA (LncRNA-LET) also affects the functions of GCs (proliferation, migration, and EMT) by affecting traditional signaling pathways (Wnt signaling pathway). The role of individual lncRNA in normal GCs was summarized in Fig. 1.

## LncRNAs in PCOS GCs

### High-throughput studies of lncRNAs in PCOS GCs

Polycystic ovary syndrome (PCOS) is the most common cause of anovulatory infertility in women, mainly manifesting polycystic ovary and hyperandrogenism. The potential role of lncRNAs in the pathogenesis of PCOS was studied in human primary GCs and a GCs cell line, human granulosa-like tumor cell line (KGN). Microarrays were performed to compare “lncRNAome” in primary GCs obtained from seven PCOS patients and seven control women. The primary human GCs were mainly cumulus GCs. In total, 862 lncRNAs and 998 mRNAs were differentially expressed in the PCOS GCs. Real-time reverse transcription PCR (qRT-PCR) was used to validate lncRNA HCG26 expression in GCs obtained from 53 PCOS patients and 50 controls. HCG26 expression was up-regulated in PCOS patients and positively correlated with follicle number at a mature stage. HCG26 knockdown in KGN cells inhibited cell proliferation and cell-cycle progression. In addition, aromatase gene (CYP19A1) expression and estradiol production were also increased in HCG26-knockdown KGN cells. This study was the first to report the lncRNA profiles in GCs obtained from PCOS patients, and suggested that dysregulated lncRNAs might play vital roles in GCs proliferation and steroidogenesis [41].

Another high-throughput study showed that 623 lncRNAs and 260 mRNAs were significantly altered and these differences could be used to discriminate GCs from PCOS patients and normal controls. Interestingly, some differentially expressed lncRNAs that are transcribed from chromosome 2 could act as enhancers, regulating neighboring coding genes. Therefore, we compared all differentially expressed lncRNAs that are transcribed from chromosome 2 and enhancer-like lncRNAs that were differently expressed in PCOS GCs. Fourteen overlapping lncRNAs were found among these two lncRNA sets (full list in supplementary Table 1). The relationship between these enhancer-like lncRNAs and their neighboring coding genes in GCs requires further investigation. In addition, among all the significantly up- or down-regulated lncRNAs and mRNAs, 43 lncRNAs and 29 mRNAs were closely correlated to construct “coding and non-coding gene networks.” Most lncRNA-mRNA pairs correlated positively. These results showed that lncRNAs are aberrantly expressed in GCs of PCOS patients. Dysfunction of these lncRNAs might account for the occurrence of PCOS and affect oocyte development [42].

According to the guidelines of both the Rotterdam or National Institutes of Health, hyperandrogenism is one of the most important diagnostic criteria of PCOS [43]. Hyperandrogenism, abnormal follicular development and ovulation largely result from GCs dysfunction [44]. LncRNAs were profiled in GCs that isolated from 4 women with PCOS with hyperandrogenism (PCOS-T) or without hyperandrogenism (PCOS-N) and healthy controls. Compared to the control group and PCOS-N, 3000 and 1030 lncRNAs were significantly altered in PCOS-T GCs (≥ two-fold change). In addition, a total of 1361 differentially expressed lncRNAs were detected in PCOS-N compared to the control group. Corticotropin releasing hormone binding protein (CRHBP) was the largest up-regulated lncRNA in PCOS-T compared to either PCOS-N or control. GO and KEGG pathway analysis suggested that CRHBP plays an essential role in mitochondrial function by interacting with transcription factors such as YY1 and SIX5 in PCOS-T GCs. This group of lncRNAs, represented by CHBRP, may play a key role in the steroid genesis and metabolism in PCOS [45].

### Indvidual studies of lncRNAs in PCOS GCs

#### LncRNA-SRA

The levels of lncRNA steroid receptor RNA activator (SRA) had increased in the peripheral blood of PCOS patients. SRA overexpression promoted GCs (the isolated primary mouse GCs are mixed cumulus GCs and mural GCs) growth and regulated the cell cycle by elevating several cell-cycle associated proteins, including Cyclin B, Cyclin E, and Cyclin D1. SRA also inhibited cell apoptosis by increasing the ratio of bcl-2:bax and repressing the levels of cleaved-caspase 3 and cleaved-Poly (ADP-Ribose) polymerase (PARP). In addition, SRA elevated the production of estradiol and progesterone, and that of two key enzymes, CYP19A1 and CYP11A1, in GCs, suggesting that SRA is a potential risk factor for PCOS [46]. A follow-up study on SRA from the same group further showed that SRA promoted ovary injury and the production of angiogenic factors in a mouse model of PCOS. Moreover, SRA increased the production of pro-inflammatory cytokines and stimulated NF-κB nuclear translocation in primary GCs obtained from PCOS mice. This study validated the important role of SRA in the development of PCOS [47].

#### LncRNA-PWRN2

Prader-Willi region non-protein coding RNA 2 (PWRN2) expression was associated with oocyte nuclear maturation in PCOS patients. In cumulus GCs, PWRN2 regulates 176 lncRNAs and 131 mRNAs. Based on these microarray data, a PWRN2-miR-92b-3p-transmembrane protein 120B (TMEM120B) competing endogenous RNA (ceRNA) network was established. This network was further validated by a luciferase report assay that revealed that miR-92b-3p directly binds to PWRN2 and TMEM120B. Therefore, PWRN2 is involved in oocyte nuclear maturation in PCOS patients by acting as a ceRNA [48]. Interestingly, PWRN2 was also identified as a significantly up-regulated lncRNA in genomic screening of another group of PCOS patients [42], validating that PWRN2 is indeed an essential lncRNA in a larger cohort of PCOS patients.

#### LncRNA-BANCR

BRAF-activated non-protein coding RNA (BANCR) is a well-studied lncRNA that plays pivotal roles in various malignancies, such as melanoma [49] and endometrial cancer [50]. BANCR acts as an “onco-lncRNA” via interactions with ERK/MAPK pathways. BANCR expression was significantly higher in cumulus GCs obtained from PCOS patients. Insulin treatment up-regulated BANCR expression in primary cumulus GCs and in the GCs line, KGN. BANCR overexpression repressed KGN cell proliferation and induced KGN cell apoptosis by promoting two pro-apoptotic markers, Bax and p53, suggesting that BANCR participates in PCOS by promoting cumulus GC apoptosis [51].

#### LncRNA- LINC-01572:28

In human primary cumulus GCs obtained from PCOS patients a negative association was found between LncRNA LINC-01572:28/p27 protein and PCNA. Also, there was a positive correlation between LINC-01572:28 expression and testosterone secretion from primary cumulus GCs. Furthermore, LINC-01572:28 inhibited cumulus GCs proliferation and prevented G1/S transition, which were partially reversed by p27 knockdown. This indicated that LINC-01572:28 suppressed cumulus GCs proliferation and cell cycle progression by reducing the degradation of p27 protein via competitive binding to S-phase kinase associated protein 2 (SKP2)-a key component of the SCF-SKP2 ubiquitin-ligase complex, which mediates cyclin E/CDK2-dependent ubiquitination and degradation of p27 [52].

#### LncRNA-HUPCOS

Androgen overdose is a key feature of PCOS. Researchers used microarrays to measure the profile of the abnormally expressed lncRNAs in GCs from 8 PCOS patients. A very highly expressed lncRNA in PCOS GCs was named as HUPCOS, which was positively correlated with follicle testosterone in PCOS patients. HUPCOS knockdown increased aromatase expression, which also promoted the conversion of androgens to estrogen. In addition, RNA-binding protein with multiple splicing RBPMS was identified as a direct binding protein of HUPCOS. This study showed that a novel lncRNA, HUPCOS, mediated androgen excess in the follicular fluid of PCOS patients by inhibiting aromatase expression via interaction with RBPMS [53].

#### LncRNA-PVT1

Liu et al. studied the role of lncRNA-PVT1/miR-17-5p/PTEN axis in PCOS ovarian GCs. Binding between PVT1 and miR-17-5p and the targeting between miR-17-5p and PTEN are determined by bioinformatics analysis, luciferase activity assay, RNA-induced silencing complex assay, and RNA pull-assay. PVT1 and PTEN were both highly expressed in PCOS ovarian GCs and follicular fluid, whereas miR-17-5p was not. MiR-17-5p overexpression and PVT1 knockdown could slow apoptosis and promote the cloning formation and proliferation of ovarian GCs in PCOS. In addition, PVT1 overexpression and miR-17-5p inhibition reversed these results. This study showed that both PVT1 down-regulation and miR-17-5p up-regulation lead to PTEN inhibition, which promoting proliferation and repressing apoptosis of ovarian GCs in PCOS [54].

#### LncRNA-TUG1

LncRNA TUG1 was significantly high in PCOS GCs and was associated with the antral follicle count. TUG1 was mainly expressed in GC nucleus. TUG1 knockdown in KGN cells inhibited proliferation and promoted apoptosis of GCs. TUG1 knockdown led to increased expression of bax, bak, cleaved caspase-3, caspase-9, cleaved caspase-9, LC3B and p-ERK proteins and decreased expression of bcl-2 and p62 proteins. In addition, using the ERK/MAPK pathway inhibitor U0126, the TUG1 knockdown-induced change in KGN cells was partially restored. Therefore, TUG1 was significantly higher in the PCOS group than that in the control group, TUG1 may inhibit cell apoptosis and autophagy in GCs through ERK/MAPK pathway inhibition and contribute to excess antral follicles. TUG1 has potential diagnostic value in PCOS [55].

**Fig. 2:**
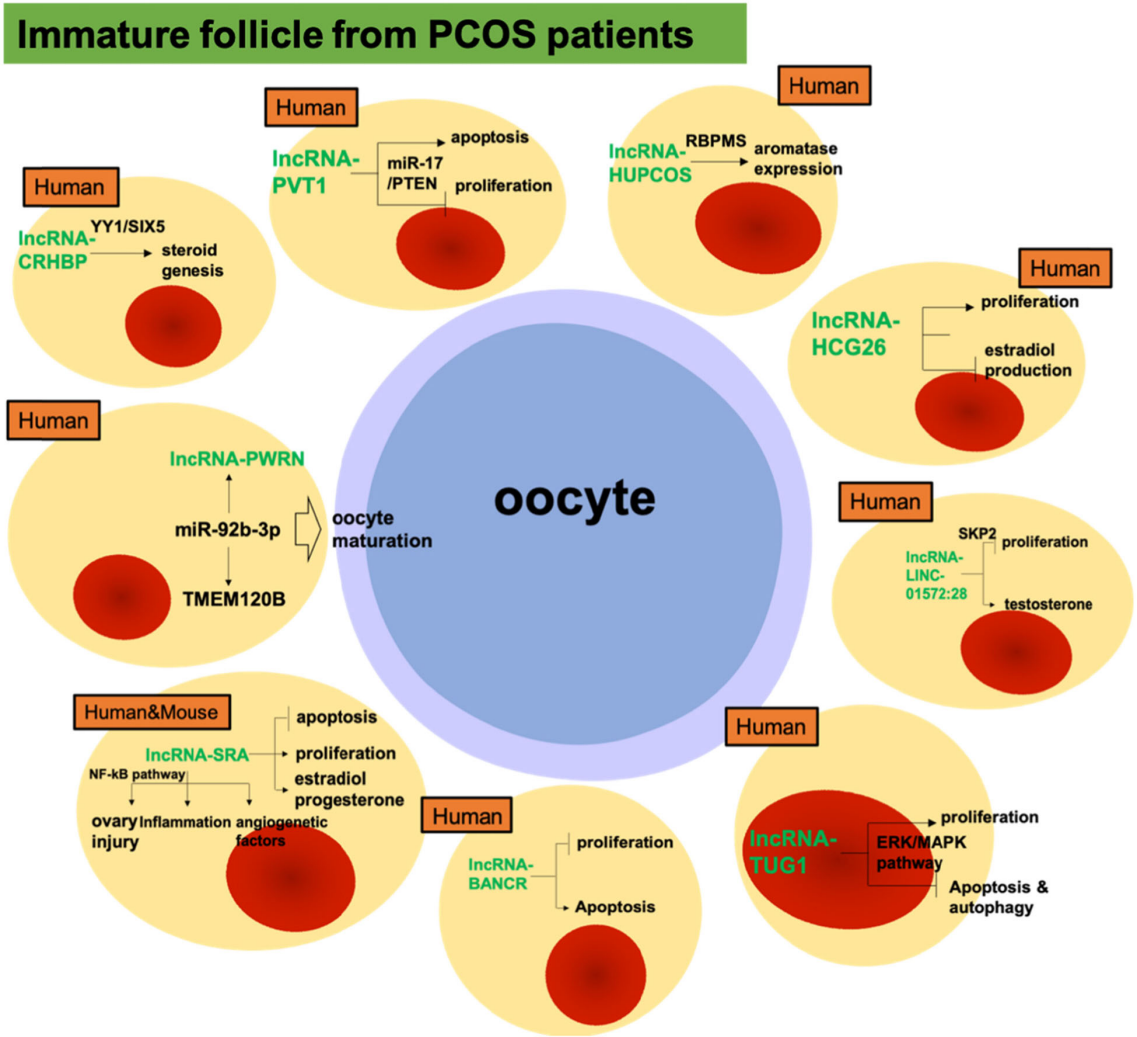
lncRNAs in GCs obtained from polycystic ovary syndrome (PCOS) patients. SRA is a potential risk factor for PCOS as it affects several functions of mural and cumulus GCs, including proliferation, apoptosis, inflammation, and production of angiogenic factors and hormones; PWNR2 is involved in oocyte nuclear maturation in PCOS by functioning as a ceRNA in cumulus GCs; BANCR participates in PCOS progression by promoting cumulus GCs apoptosis; LINC-01572:28 suppresses PCOS cumulus GCs proliferation and cell cycle progression via competitive binding to SKP2; TUG1 inhibits apoptosis and autophagy in PCOS GCs through inhibition of ERK/MAPK pathway; HUPCOS mediates androgen excess in follicular fluid of PCOS patients via interaction with RBPMS; CHBRP involves in steroid genesis and metabolism in PCOS GCs by interacting with transcription factors YY1 and SIX5; HCG26 knockdown in GCs inhibits cell proliferation induces estradiol production. PVT1 down-regulation and miR-17-5p up-regulation lead to PTEN inhibition, which promoting proliferation and repressing apoptosis of PCOS GCs

Taken together, these studies suggested that GC-specific lncRNAs are involved in PCOS, which paves a novel pathway for better understanding of the molecular mechanisms underlying this reproductive disease. However, these high-throughput experiments of lncRNA only identified GC-specific lncRNAs at the transcriptome level (Table 1). To identify potential specific diagnostic markers and therapeutic targets, we need to study the specific function of individual lncRNAs in GCs from patients with PCOS. Until now, nine individual lncRNAs have been identified in PCOS GCs (Fig. 2), most of which are involved in two main cellular functions of GCs: proliferation and hormone production. Synergistic effects between lncRNAs with similar functions should be further investigated in the future.

**Table 1:**
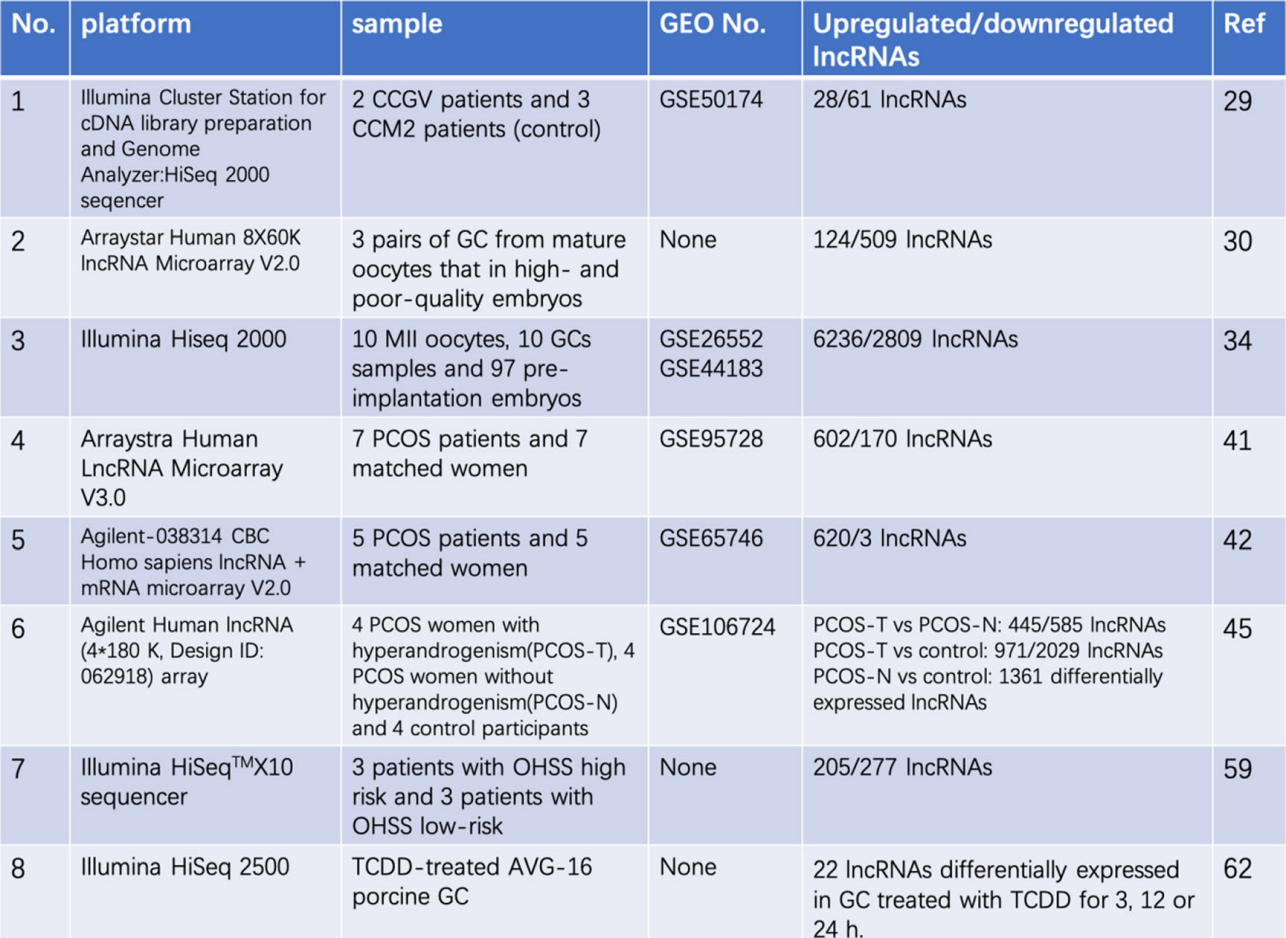
LncRNA expression profiling in granulosa cells (GCs)

## The role lncRNAs in POI GCs

Premature ovarian insufficiency (POI, also referred to as premature ovarian failure [POF]) is a common cause of female infertility. GC apoptosis is one important mechanism underlying this decline in ovarian function [56]. Cyclophosphamide treatment was found to repress GCs growth and lead to ovarian atrophy. Northern blot results demonstrated that the intensity of the lncRNA-Meg3 hybridization signal was up-regulated in cyclophosphamide-treated GCs (the isolated primary mouse GCs are mixed cumulus GCs and mural GCs). Expressions of apoptotic markers, including p53, p66Shc, p16, and cleaved caspase-3, were all decreased in lncRNA-Meg3-knockdown GCs compared to control. Thus, cyclophosphamide deferred GCs proliferation and promoted POF by inducing lncRNA-Meg3 [23].

Wang et al. found lncRNA HCP5 decreased in GCs that isolated from patients with biochemical POI (patients with normal menstrual cycle and increased gonadotropin). HCP5 stabilized the interaction between YB1 and ILF2, mediating the YB1 transfer into GC nucleus. HCP5 knockdown affected this transfer and reduced its binding to to the promoter region of MSH5 gene and repressing its transcription. Reduced HCP5 expression in GCs of biochemical POI patients contributed to the generation of dysfunctional GCs by modulating MSH5 transcription via the interaction with YB1. This study showed a novel epigenetic mechanism for POI pathogenesis [57].

To investigate whether lncRNA-FMR6 is involved in the development of fragile X-associated premature ovarian insufficiency (FXPOI), 22 consecutive fragile X mental retardation 1 (FMR1) premutation carriers undergoing IVF and pre-implantation genetic diagnosis (IVF-PGD) were studied. Eleven patients undergoing IVF- intracytoplasmic sperm injection (ICSI) were recruited as a control group and their cumulus GCs were isolated after oocyte retrieval. In FMR1 premutation carriers, there was a nonlinear association between the number of CGG repeats and the transcriptional level of lncRNA-FMR6. A negative linear correlation was observed between the number of oocytes retrieved and lncRNA-FMR6 expression in cumulus GCs, indicating that the RNA toxic gain-of-function is a potential pathophysiological mechanism of FXPOI [58].

**Fig. 3:**
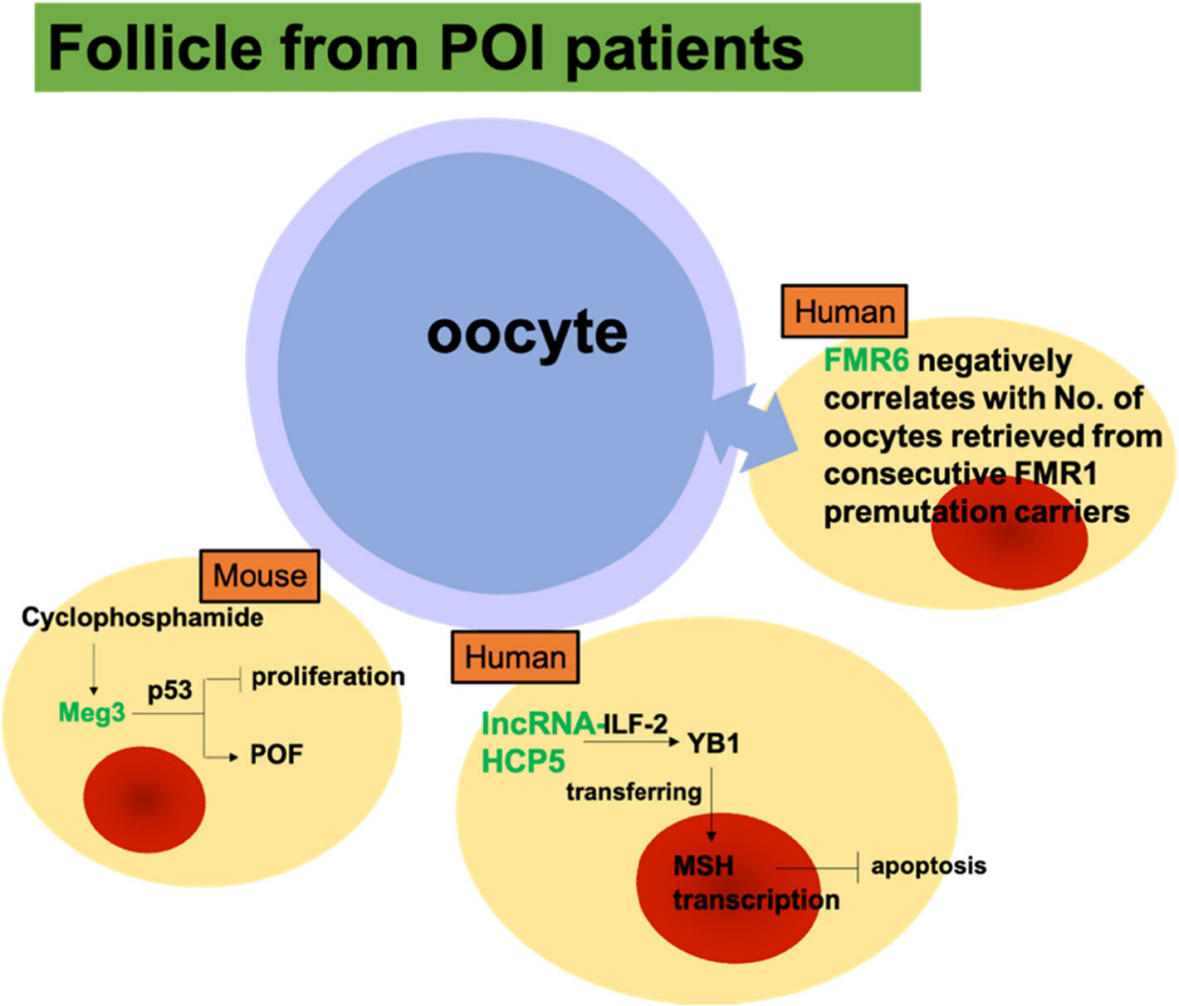
LncRNAs in GCs obtained from Premature ovarian failure (POI) patients. Cyclophosphamide defers mural and cumulus GCs proliferation and promotes POF by inducing lncRNA-Meg3; FMR6 correlates negatively with the number of oocytes retrieved from consecutive FMR1 premutation carriers in cumulus GCs; HCP5 contributed to dysfunctional GCs by modulating MSH5 transcription via the interaction with YB1

Ovarian reserves depend primarily on the number and quality of oocytes. Initially, insufficient or accelerated depletion of the primordial follicle pool will result in ovary insufficiency. It has been determined that mammalian GCs significantly contribute to the development of follicles (39, 40). In addition, reduced GCs induced follicle atresia, which eventually lead to POI (18, 19). So far, the main research field of POI pathogenesis was protein-coding genes (4–6). However, the causing-gene mutations can only explain ~ 15% of POI cases (35, 36). There is currently a lack of research on the role of individual lncRNA in POI GCs (only three reports until now, as depicted in Fig. 3). In the future, lncRNAs sequencing in POI GCs should be performed to better understand the role of lncRNAs in POI from a panoramic view, which will explore unidentified essential individual lncRNA role in POI.

## The role of lncRNAs in other GC-related diseases

To identify differentially expressed lncRNAs in ovarian hyperstimulation syndrome (OHSS), which is characterized by enlarged ovaries and up-regulated vascular permeability, GCs were obtained from women with varying OHSS risks, for high-throughput sequencing. A total of 23,815 lncRNAs were detected and 482 of them showed differential expression: 205 lncRNAs were up-regulated and 277 lncRNAs were downregulated. Several ovarian biological processes were significantly involved in this phenomenon as demonstrated by KEGG pathway and GO analyses. Meanwhile, an lncRNA/miRNA interacting network was also established according to ceRNA regulatory mechanisms, a recent finding that adds to the complexities of miRNA-mediated gene modulation. ceRNAs are RNAs that mutually regulate other miRNAs via competitively binding the same miRNA recognition elements (MREs). In addition, expression screening identified eight novel lncRNAs (Supplementary Table 2) in GCs that were associated with risk factors for OHSS, suggesting that these lncRNAs might be potential participants in OHSS development [59].

The environmental contaminant, 2,3,7,8-tetrachlorodibenzo-p-dioxin (TCDD), causes reproductive defects, such as anovulation and disorder of follicular steroidogenesis, in several mammals, including human, mouse, rat and pig [60, 61]. In total, 1666 lncRNAs were characterized in TCDD-treated porcine GCs in vitro and 22 differentially expressed lncRNAs were identified. The potential functions of these 22 lncRNAs were predicted by analyzing their *cis*- and *trans*-regulated protein-coding genes. Two essential functional proteins of GCs, cytochrome P450 1A1 (CYP1A1) and aryl hydrocarbon receptor (AhR), were identified among the genes that were *trans*-regulated by the differentially expressed lncRNAs. ﻿The mRNA-lncRNA co-expression analysis showed that the TCDD-regulated lncRNAs might be involved in numerous cellular activities in GCs, such as cellular response to xenobiotics, proliferation, and dioxin metabolism. Therefore, these GC-specific lncRNAs may be involved in TCDD-induced reproductive defects [62].

Endometriosis and its surgical treatment have an adverse effect on the ovarian reserve and on oocyte development. Metastasis associated lung adenocarcinoma transcript 1 (MALAT1), also known as nuclear-enriched abundant transcript 2 (NEAT2), is a well-studied lncRNA that is highly evolutionarily conserved [63] and is extensively investigated as an “onco-lncRNA” in various malignancies [64, 65]. Its expression was downregulated in GCs (the isolated primary mouse GCs are mixed cumulus GCs and mural GCs) from patients with endometriosis. Moreover, MALAT1-knockdown repressed GCs proliferation via the induction of the extracellular signal-regulated kinase (ERK)/mitogen-activated protein kinase (MAPK) pathway, suggesting that the MALAT1 dysfunction might have an adverse effect on the development of oocytes in endometriosis [66].

Taken together, these data reflect the important role of GC-specific lncRNAs in a series of GC-related reproductive diseases, suggesting that GC-specific lncRNAs may be used as diagnostic markers or potential therapeutic targets for these diseases. However, the underlying mechanisms have not been systematically illuminated and these studies used in vitro assays. Therefore, to elucidate the specific function and mechanism of these lncRNAs in GCs obtained from patients suffering from ovarian diseases, advanced animal models and a large number of clinical samples will be required in subsequent studies.

## LncRNAs as targets for diagnosis and treatment of GC-related diseases

To investigate the potential application of lncRNAs in clinical diagnosis and therapy of GC-related diseases, researchers currently focus on the function and underlying mechanisms of lncRNAs in GCs. High-throughput technologies, such as lncRNA sequencing, have been applied to screen lncRNAs in GCs in several pathological conditions. These differentially expressed lncRNAs may be useful for the diagnosis or prognosis of GC-associated diseases. Moreover, studies of individual lncRNAs in GCs highlight the potential use of lncRNAs as therapeutic targets for GC-related diseases. For example, encapsulated siRNA may be delivered to target specific lncRNAs for the treatment of GC-related diseases. Due to the complex spatial structure and unclear molecular regulatory mechanisms, studies of lncRNAs are still at a very early stage. Therefore, a deeper understanding of lncRNA functions in GCs will provide a promising foundation for a potential future use of lncRNAs in the diagnosis or treatment of GC-related diseases.

## Conclusion and future perspectives

Granulosa cells are widely known for playing an essential role in both normal folliculogenesis and various ovarian disorders. LncRNAs exert their effects in GCs via multiple mechanisms. Until now, all regulatory mechanisms of lncRNA in GCs could be divided into five categories: 1) lncRNAs that regulate classical signaling pathways, such as lncRNA-LET (which induces the Wnt/β-catenin/Notch pathways), or lncRNA-SRA (which activates the NF-κB pathway) and lncRNA-MALAT1 and lncRNA-TUG1(which represses the ERK/MARK pathway) in GCs; 2) lncRNAs that activate transcription via activating their host genes or adjacent coding genes in GCs, including lncRNA-Amhr2, which activates the transcription of its neighboring gene Amhr2, or lncRNA-lncPrepþ96kb, which promotes its adjacent coding gene POP and HAS2, which was activated by its antisense lncRNA-HAS2-AS1; 3) ceRNA mechanism in GCs: PWRN2-miR-92b-3p-TMEM120B ceRNA network, LncRNA-LINC-01572:28/p27-SKP2-p27 ceRNA network and PVT1-miR-17-5p-PTEN ceRNA network in GCs; 4) P53-associated lncRNAs: lncRNA-BANCR and LncRNA-Meg3; 5) transcriptional factors-associated lncRNAs: lncRNA-HUPCOS interacts with RBPMS, lncRNA-CHBRP interacts with YY1 and SIX5 and lncRNA-HCP5 interacts with YB1(Table 2). The specific functional roles, upstream regulators, and downstream effectors of these dysregulated lncRNAs in GCs are still elusive. The dysregulated expression of lncRNAs in the circulation has been used as a biomarker in several studies that allowed distinguishing patients with ovarian disorders from healthy controls; however, the clinical usage of lncRNAs for ovarian disorder diagnosis remains to be established through large-cohort validation in different populations. In the future, advanced technologies, such as single-cell sequencing, CRISPR-Cas9, genetic animal models, and subsequent clinical trials must be employed to further elucidate the mechanisms and verify the clinical potential of lncRNAs in GCs as diagnostic or therapeutic targets for ovarian disorders.

**Table 2:**
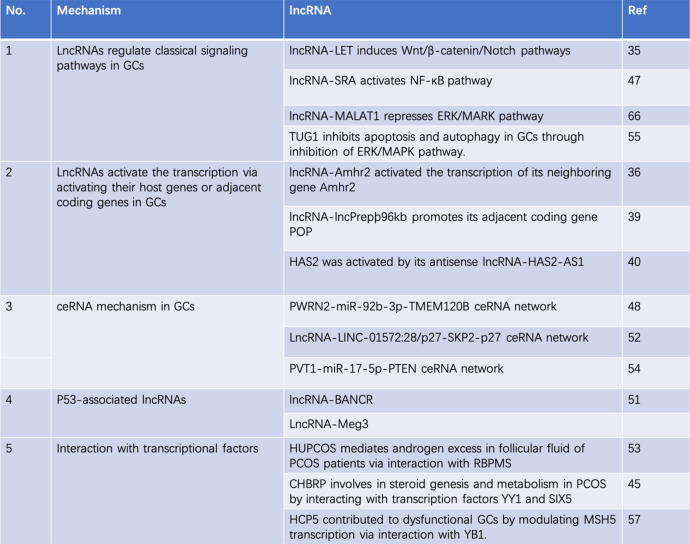
Summary of regulatory mechanisms of lncRNAs in granulosa cells (GCs)

## Supplementary information

**Additional file 1.** The fourteen overlapping lncRNAs that are transcribed from chromosome 2 and enhancer-like lncRNAs that were differently expressed in PCOS GCs.

**Additional file 2.** The eight novel lncRNAs in GCs that were associated with risk factors for OHSS.

## Data Availability

Not applicable.
